# Comment on Alquraini et al.: reliability of Canadian Emergency Department Triage and Acuity Scale (CTAS) in Saudi Arabia

**DOI:** 10.1186/s12245-015-0090-3

**Published:** 2015-11-11

**Authors:** Amir Mirhaghi

**Affiliations:** Evidence-Based Caring Research Center, Department of Medical-Surgical Nursing, School of Nursing and Midwifery, Mashhad University of Medical Sciences, Mashhad, Iran; Department of Medical-Surgical Nursing, School of Nursing and Midwifery, Chahrrah-e-Doktorha, Mashhad, Khorasan Razavi Iran

**Keywords:** Kappa, Agreement, Reliability, Triage, Emergency

## Abstract

It is very common to examine reliability of triage scales using (weighted) kappa statistics. The point is that weighted kappa has grossly underestimated disagreements by one category and put more emphasis on extreme category disagreements; therefore, low prevalence of critically-ill and non-urgent patients has excluded the effect of extreme categories disagreement from calculated kappa coefficient and also contributed to significant overestimation. As a result, weighted kappa coefficient as an estimate of scale reliability is overestimated by the anchoring effect.

## Dear editor

With great interest, we have read the outstanding publication in the recent International Journal of Emergency Medicine from Alquraini et al. entitled “Reliability of Canadian Emergency Department Triage and Acuity Scale (CTAS) in Saudi Arabia” [1].

The Canadian Triage and Acuity Score (CTAS) has been structured using narrative description of the patient’s medical condition including alarming signs and symptoms for each high-risk complaint [2]. However, narrative triage scales let nurses to decide much more freely than they do by algorithm-based scales such as Emergency Severity Index (ESI) [3], and also, the CTAS does not force nurses to allocate patients into any particular category [4]; it should be noted that it may affect triage concordance and lessen agreement among triage nurses instead.

We would like to bring your attention to the discrepancy concerning agreement across the extreme categories. Raw agreement among nurses has been reported 52.31 % for resuscitation (CTAS I), 55.63 % emergent (CTAS II), 61.13 % urgent (CTAS III), 53.85 % less urgent (CTAS IV), and 56.14 % for non-urgent (CTAS V) level [1], so it means there was concordance among categories only in half of the cases. It has also resulted in kappa value of 0.871 indicating good agreement [1]. Whereas, generally, there is an expected agreement on extreme categories more than twice as much as other categories [4]. The vital question is that if the kappa coefficient represents the extent to which reliability exists.

Two main issues must be clearly addressed to answer this question. First of all, it has been clarified that most of the cases triaged as urgent and less urgent (CTAS levels III and IV) [1], so it means ED census has provided certain type of patients for nurses to be triaged especially level III and IV patients. Existing awareness of high prevalence of urgent patients will lead toward a particular decision, and the triage nurses tended to quickly focus on urgent patients; it leads to completely wildly optimistic results and unrealistic substantial kappa value. The anchoring effects can occur, while nurses are less likely to choose a low-end or end-point of triage scale. Anchoring effects are almost always present as there is usually a less extreme way to label the high and low ends of a scale. Anchoring effect is a fully recognized bias in which people start with an implicitly suggested reference point (the “anchor”) and make adjustments to it to reach their estimate. People start with an initial estimation (anchor) and after that make further adjustments based on additional information. Insufficient adjustments often give the initial anchor a great deal of influence over future assessments [5].

In addition, weighted kappa has grossly underestimated disagreements by one category and put more emphasis on extreme category disagreements [6]; therefore, low prevalence of critically-ill and non-urgent patients has excluded the effect of extreme categories disagreement from calculated kappa coefficient and also contributed to current results either. As a result, weighted kappa coefficient as an estimate of scale reliability is overestimated by the anchoring effect. Overall, it has been suggested that the results should be interpreted with extreme cautious.

We highly recommend further studies on the reliability of the five-level triage scales and desired outcomes including time-related indices, mistriage rates (over-triage and under-triage), weighted and un-weighted kappa coefficients concerning different populations, and emergency clinicians in Saudi Arabia.

## Abbreviations

CTAS: The Canadian Triage and Acuity Score
ESI: Emergency severity index
